# Gold and Amalgam as a Basis for Filling

**Published:** 1893-03

**Authors:** W. F. Simonton

**Affiliations:** Cameron, W. Va.


### GOLD AND AMALGAM AS A BASIS FOR FILLING.

BY DR. W. F. SIMONTON, CAMERON, W. VA.

Approximal cavities of molars and bicuspids give more trouble, and the fillings fail oftener, than any other class. The following method of treating approximal cavities of molars and bicuspids has given me better results than any other. It is, or might be called, a modification of the method of filling partly with amalgam, and letting it harden, and finishing the filling with gold at a future sitting, or of Dr. Clapp's method,—of finishing all at one sitting.

Let us consider, for instance, a distal cavity in the first molar, and a mesial cavity in the second, perhaps as difficult an operation of the kind as any dentist will be called upon to perform. First, secure sufficient space by pressure or cutting, or both, but this must be obtained to allow the operator to see all parts to be operated upon. Prepare the cavity by cutting back the walls, if possible, until some enamel is reached, especial attention being given to the lower or cervical wall; cut it down until it is smooth and strong, with an abutment of dentine back of it, and do this even if it carries the cervical wall below the gum margin, and do not trust, if it can at all be avoided, a thin bevelled cervical wall. Cut a retaining-groove across the cervical wall or bottom of the cavity, letting it extend up the sides out through the coronal enamel, if a coronal opening has been given to it. At the ends of the groove across the floor, where it turns up the sides, make the angles somewhat acute or with slight pits; and the groove across the floor should be far enough from the enamel to leave standing a small abutment of dentine between the groove and the enamel. Prepare a small piece of amalgam, well washed with alcohol and pressed dry with pliers, and place in the cavity, to fill or nearly fill the retaining-groove, but be careful to keep the amalgam back from the enamel or outer edge of cavity at the cervical border. Now place on the amalgam small pieces of gold, not pellets, but foil folded and cut in very small squares. I use Williams's crystalloid, No. 3, cut in pieces a line or less square, or small bits of Stuerer's or Watt's crystal. Press these first pieces down lightly, using a small instrument, and applying it all over the gold; and soon the mercury will show through the gold; then apply more gold, letting it extend to and beyond the cervical edge of the cavity.

It may be well here to diverge somewhat to speak of cavities where it is impossible to leave an abutment of dentine at the bottom, because the dentine may be decayed below perfect enamel, and the decay extend out to the enamel at the cervical wall. This will give a pocket- or pouch-shaped space alongside the enamel at the cervical border. If so, be careful not to fill this pocket full of amalgam, the idea being to have only enough to furnish mercury to amalgamate the first few layers of gold next the cervical border. After the mercury ceases to show through, press the gold down firmly, packing it in the angles or pits at end of lower retaining-groove, which will be found to hold this part of the filling securely. The filling will now be about the eighth of an inch above the cervical wall, and firmly held by the ends of the retaining-groove. Proceed now to condense and finish, first using pressure by means of a burnisher or any suitable instrument, afterwards the mallet, if thought necessary; but pressure is generally sufficient, and it avoids danger of shattering or crumbling this wall. Start the filling in both cavities before completing either, but commence the filling in the distal cavity, as it is more convenient, and complete the filling in the mesial cavity first, as a portion of the front teeth being removed gives much better access to the mesial cavity and also admits the light, while if the mesial filling is completed and partly finished, it acts as a reflector on the distal cavity, and makes it much lighter during the filling.

By following this method and not using any matrix, we get an amalgam or substance for filling the cervical portion of the cavity that is soft and tough and easily adapted to the enamel edge. Be sure that this important part of the work must be well done, and no overlapping of the filling left at the cervical wall. This opportunity to finish will be more appreciated by recollecting the difficulty at the cervical border, where the teeth are long, or the filling extends to or under the margin of the gum, or the neck of the tooth is small, and that portion of the tooth next the gum slopes from a large coronal surface to meet a narrow neck. In short, the method tries, at least, to take into consideration the fact that these fillings almost always fail first at the cervical border, and are also most difficult to make properly here. The use of the matrix cuts off all light at the cervical border. The enamel may be injured by the first few blows of the mallet, or a small portion be scaled off, but the operator knows nothing or little of this after the filling is commenced.

If it be desired, a matrix may at this point be applied for finishing the filling. This method of starting a filling and of finishing the cervical portion is often useful in approximal cavities of incisors, whether there be only one or two fillings that face each other; it is often an excellent plan to put in the cervical part of the filling and at least roughly trim off the surplus gold, as we get much better access to and can see the cervical part more clearly before the main body of the filling is inserted.

